# 0036. Confocal imaging of impaired mitochondrial function in the cerebral cortex of rats during haemorrhagic shock *in vivo*

**DOI:** 10.1186/2197-425X-2-S1-O9

**Published:** 2014-09-26

**Authors:** KK Ida, LMS Malbouisson, DA Otsuki, KI Chisholm, A Dyson, M Singer, MR Duchen, KJ Smith

**Affiliations:** University of São Paulo, Medical School, Anaesthesiology LIM-8, São Paulo, Brazil; University College London, Department of Neuroinflammation, Institute of Neurology, London, UK; University College London, Bloomsbury Institute of Intensive Care Medicine, London, UK; University College London, Department of Cell and Developmental Biology, London, UK

## Introduction

Haemorrhagic shock (HS)-induced hypotension impairs cerebral perfusion pressure and decreases oxygen supply to the brain (1), but the consequences for brain mitochondrial function are poorly understood.

## Objectives

To investigate the effects of HS on mitochondrial function in the cerebral cortex of rats *in vivo*.

## Methods

A flap of skull was removed and cerebral cortex exposed in isoflurane-anaesthetised rats undergoing HS (target mean arterial pressure (MAP) of 40 mmHg for 30 minutes) (*n*=7) compared with sham (*n*=6) controls. Mitochondrial function was assessed by observing mitochondrial membrane potential (using tetramethyl rhodamine methyl ester (TMRM); ex: 543 nm; em: 585 nm), and endogenous flavin adenine dinucleotide (FAD) autofluorescence (ex: 488 nm; em: 505-570 nm) by confocal imaging. The TMRM and FAD signals were registered before induction of HS (baseline), at MAP of 40 mmHg (shock), and at 30 (T30) and 60 (T60) minutes after shock.

## Results

**Figure 1:**
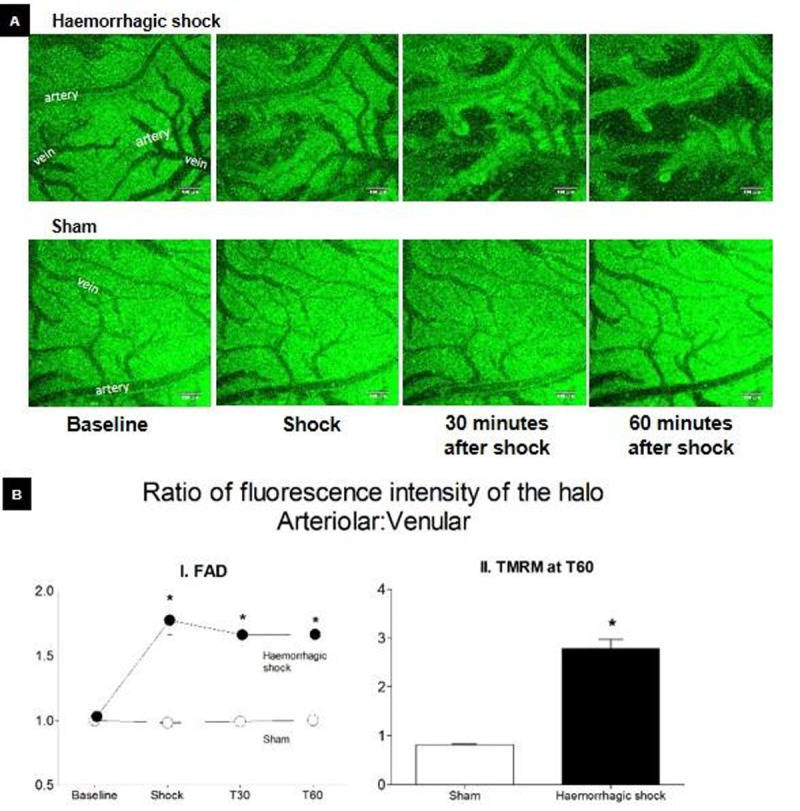
(A) Images of the somatosensory cortex at baseline, haemorrhagic shock, and at 30 and 60 minutes after shock. Green mitochondrial FAD autofluorescence indicates an oxygenated environment, and in shick this is only maintained within ~30-40 μm of arteries. (B) Ratio of the FAD (I) and TMRM (II) fluorescence intensity of the halo extending 30-40 μm, from arterioles/arteries to venules/veins in rats undergoing haemorrhagic shock. *Within a time-point, the value is significantly different from sham (*p* <0.01).

There was a loss of mitochondrial membrane potential (decreased TMRM signal; *p* < 0.001) and reduction of the FAD/FADH_2_ pair (seen as decreased FAD fluorescence; *p* < 0.01) at both T30 and T60 post-shock, except for a striking protection observed as a 'halo' extending ~30-40 mm from arterioles (Figure 1). No loss of mitochondrial function was observed in sham controls (MAP >90 mmHg in all animals at both T30 and T60).

## Conclusions

HS-induced hypotension causes a marked loss of mitochondrial membrane potential and FAD fluorescence in cerebral cortex, except in regions in immediate proximity to arterioles. This study reveals a profound spatial vulnerability of cortical tissue to reduced blood flow.

## References

[1] Cavus E, Meybohm P, Doerges V (2009). Cerebral effects of three resuscitation protocols in uncontrolled haemorrhagic shock: a randomised controlled experimental study. Resuscitation.

